# Mechanisms Underlying the Confined Diffusion of Cholera Toxin B-Subunit in Intact Cell Membranes

**DOI:** 10.1371/journal.pone.0034923

**Published:** 2012-04-12

**Authors:** Charles A. Day, Anne K. Kenworthy

**Affiliations:** 1 Department of Molecular Physiology and Biophysics, Vanderbilt University School of Medicine, Nashville, Tennessee, United States of America; 2 Department of Cell and Developmental Biology, Vanderbilt University School of Medicine, Nashville, Tennessee, United States of America; 3 Chemical and Physical Biology Program, Vanderbilt University School of Medicine, Nashville, Tennessee, United States of America; Institut Curie, France

## Abstract

Multivalent glycolipid binding toxins such as cholera toxin have the capacity to cluster glycolipids, a process thought to be important for their functional uptake into cells. In contrast to the highly dynamic properties of lipid probes and many lipid-anchored proteins, the B-subunit of cholera toxin (CTxB) diffuses extremely slowly when bound to its glycolipid receptor GM_1_ in the plasma membrane of living cells. In the current study, we used confocal FRAP to examine the origins of this slow diffusion of the CTxB/GM_1_ complex at the cell surface, relative to the behavior of a representative GPI-anchored protein, transmembrane protein, and fluorescent lipid analog. We show that the diffusion of CTxB is impeded by actin- and ATP-dependent processes, but is unaffected by caveolae. At physiological temperature, the diffusion of several cell surface markers is unchanged in the presence of CTxB, suggesting that binding of CTxB to membranes does not alter the organization of the plasma membrane in a way that influences the diffusion of other molecules. Furthermore, diffusion of the B-subunit of another glycolipid-binding toxin, Shiga toxin, is significantly faster than that of CTxB, indicating that the confined diffusion of CTxB is not a simple function of its ability to cluster glycolipids. By identifying underlying mechanisms that control CTxB dynamics at the cell surface, these findings help to delineate the fundamental properties of toxin-receptor complexes in intact cell membranes.

## Introduction

The role of cholesterol-dependent membrane domains have been intensively investigated as a mechanism involved in the regulation of membrane trafficking and signaling in cells [1]. Initially envisioned to exist as stable platforms, such domains are now thought to consist of transient nanoscopic assemblies of proteins, glycolipids, and cholesterol [2]. As such, current models suggest that mechanisms that crosslink components of these domains may be important for facilitating their functions [2], as well as to alter membrane mechanics and deform membranes [3].

Bacterial toxins in the AB_5_ family, including Shiga toxin and cholera toxin, are an example of a class of proteins with the intrinsic capacity to crosslink glycolipids via their multivalent membrane binding B-subunits [4]–[11]. The ability of cholera toxin B-subunit (CTxB) and related molecules such as Shiga toxin B-subunit to cluster glycolipids and organize membrane domains has been linked to their functional uptake into cells by clathrin-independent, cholesterol-dependent endocytic pathways [3], [7], [12], [13]. Recently, it has become evident that the accessibility of glycolipids to toxin binding is itself regulated by cholesterol within both model membranes and cell membranes, as a significant fraction of glycolipids is masked and inaccessible to toxin binding [14], [15]. Thus, a picture is emerging in which the ability of toxin to bind glycolipids is controlled in a cholesterol-dependent manner [14], [15] and the presence of bound toxin itself also leads to changes in underlying membrane domain structure [3], [9]–[11], [16].

An important question raised by these findings is how the structure and dynamics of the complex formed upon binding of toxins to the accessible pool of their glycolipids receptors are regulated in cells. For the case of cholera toxin, one striking feature of the CTxB/GM_1_ complex is that it diffuses extremely slowly within the plasma membrane compared to many other proteins and lipids [13], [17]–[22]. This result is surprising given that lipids themselves typically diffuse rapidly in cell membranes, as do many lipid-anchored proteins [22]–[28]. This suggests that the movement of the CTxB/GM_1_ complex within the plasma membrane is regulated by fundamentally different mechanisms than those that control the dynamics of other types of cell surface molecules under steady state conditions.

The underlying mechanisms that contribute to the slow diffusion of CTxB are not yet fully understood. However, several factors could potentially account for this behavior. For example, there is some evidence that CTxB is confined by actin-dependent barriers [17]. CTxB could potentially associate with nanoclusters that form via an energy- and actin-dependent process, similar to those reported for other lipid-tethered proteins [29]. CTxB has also been reported to associate with caveolae [30]–[33], flask-shaped invaginations of the plasma membrane which themselves are immobilized within the plane of the membrane [34], [35]. The intrinsic ability of CTxB to cluster glycolipids could potentially lead to the formation of slowly diffusing CTxB/GM_1_ complexes. If they became large enough, such complexes could also potentially impact the diffusional mobility of other molecules, by either forming barriers to their diffusion or by trapping them within the same domains [36], [37]. In the current study, we investigated the contributions of these various factors to the confined diffusion of CTxB within the plasma membrane of living cells using confocal FRAP.

## Results

### Confocal FRAP assay and cell surface markers examined in this study

To measure the diffusion of CTxB on the plasma membrane, we took advantage of a quantitative confocal FRAP-based assay that yields accurate diffusion coefficients for both rapidly and slowly moving molecules [38], [39]. In FRAP, lateral diffusion is described by two parameters, the diffusion coefficient (*D*), reflecting the average rate of diffusion, and the mobile fraction (M_f_), a measure of fraction of molecules that are free to recover over the time course of the experiment.

**Figure 1:**
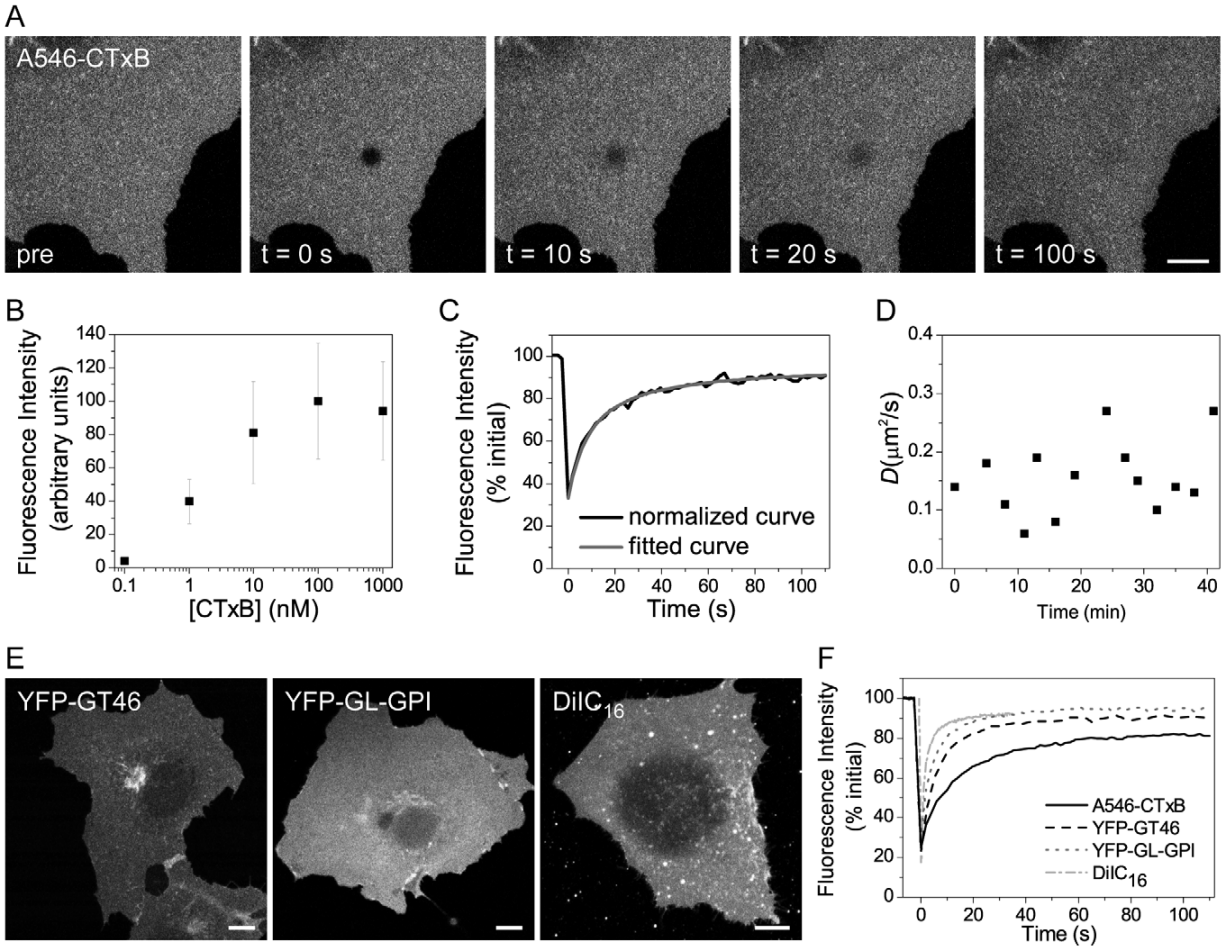
Confocal FRAP assay. COS-7 cells were transfected with the indicated constructs or left untransfected and labeled with Alexa546-CTxB or DiIC_16_. FRAP was performed at 37°C using a 4.1 µm diameter bleach spot. (**A**) Representative images of Alexa546-CTxB during a FRAP experiment. Bar, 10 µm. (**B**) Average fluorescence intensity of Alexa546-CTxB labeling of COS-7 cells incubated with A546-CTxB concentrations ranging from 0.1 nM to 1 µM (mean ± SD for 59–151 cells). (**C**) Example of a normalized recovery curve for a cell labeled with 1 µM Alexa546-CTxB after correcting for fluorescence decay during imaging, along with fitted FRAP curve. (**D**) Representative example of *D* for CTxB as a function of time after labeling. Each value of *D* was obtained for a different cell on the same coverslip from a single experiment. (**E**) Representative whole cell images of YFP-GT46, YFP-GL-GPI, and DiIC_16_ in COS-7 cells. Single confocal slices are shown. The spotty appearance of DiIC_16_ on the background is due to the presence of dye aggregates. Bar, 10 µm. (**F**) Representative FRAP curves for Alexa546-CTxB, YFP-GT46, YFP-GL-GPI, and DiIC_16_ (n = 8–13 cells for each).

To quantify the diffusional mobility of CTxB at the cell surface, COS-7 cells were labeled briefly with saturating levels of CTxB (1 µM) (Figure 1B), washed, then shifted onto the microscope stage. We visualized a portion of the plasma membrane, and FRAP measurements were carried out using a circular bleach region (Figure 1A). Although CTxB was endocytosed to the perinuclear region in a time-dependent manner, a substantial fraction of CTxB remained associated with the plasma membrane over time, enabling measurements of its cell surface mobility by confocal FRAP over at least 30 minutes after shifting cells to the microscope stage at 37°C. Care was taken to exclude any FRAP data in which non-surface attached, mobile endocytic vesicles were inside the ROI at any time during the FRAP experiment. The recovery curves were well fit by a pure diffusion model, implying that the recovery process is dominated by lateral diffusion (Figure 1C). In addition, the diffusional mobility of CTxB remained constant over time, suggesting that the properties of the cell surface pool of CTxB do not change significantly even while some of the toxin is being actively endocytosed (Figure 1D).

In order to understand what aspects of the regulation of CTxB's diffusion are specific for molecules that cluster glycolipids, we examined of the diffusional mobility of several additional cell surface markers in parallel in our study: a representative GPI-anchored protein, YFP-GL-GPI [40], a single pass transmembrane protein, YFP-GT46 [41], and a fluorescent lipid analog, DiIC_16_ (Figure 1E). Our rationale for studying these markers was severalfold. GPI-anchored proteins are linked to cell membranes via a lipid anchor, and also have been shown to associate with cholesterol-dependent nanoclusters [29], [42] that could potentially organize glycolipids as well. We chose to study YFP-GT46 because transmembrane proteins are often subjected to different types of constraints to their diffusion than are lipid-anchored proteins [23], [27]. DiIC_16_ was selected for these studies to control for the fact that CTxB binds to a lipid receptor at the cell surface. In general, lipids diffuse much more rapidly than proteins do in cell membranes [23]–[27]. Because CTxB clusters multiple glycolipids, it would not necessarily be expected to behave like a simple reporter of lipid diffusion.

Previous studies have shown that CTxB diffuses significantly more slowly than YFP-GL-GPI, YFP-GT46, and DiIC_16_ when directly compared in the same cell line [22], [28]. We confirmed this in control FRAP experiments (Figure 1F). The fastest value of *D* was measured for DiIC_16_ (2.54±0.78 µm^2^/s, mean ± SD). *D* for YFP-GL-GPI (1.18±0.49 µm^2^/s) was slower than for DiIC_16_ but faster than that of YFP-GT46 (0.54±0.18 µm^2^/s), while CTxB diffused the most slowly of all (0.17±0.12 µm^2^/s). M_f_ was ∼90% for YFP-GL-GPI, YFP-GT46, and DiIC_16_, and ∼80% for CTxB (Table S1). These data indicate the diffusion of CTxB is selectively constrained at the cell surface relative to these other classes of molecules. We next investigated possible mechanisms underlying the slow diffusion of CTxB. For the purpose of these studies, we focused on understanding the properties of CTxB bound to its accessible pool of glycolipid receptors [14], [15].

### CTxB diffusion is confined by the actin cytoskeleton

The actin cytoskeleton is a well-known barrier to the diffusion of a number of cell surface molecules [27], [43]–[46]. Two previous observations suggest that actin may play a role in controlling the diffusional mobility of CTxB. First, biochemical studies indicate that cholera toxin co-fractionates with actin [47]. Second, CTxB diffusion was shown to be enhanced following actin disruption [17]. However, the latter study did not evaluate how actin disruption affected the mobility of other proteins and lipids, leaving open the question of how specific this effect was. We therefore sought to directly compare the impact of disrupting the actin cytoskeleton on the diffusion of CTxB relative to its effect on other proteins and lipids at the cell surface using latrunculin A (LatA), which inhibits actin polymerization by sequestering monomeric actin.

**Figure 2:**
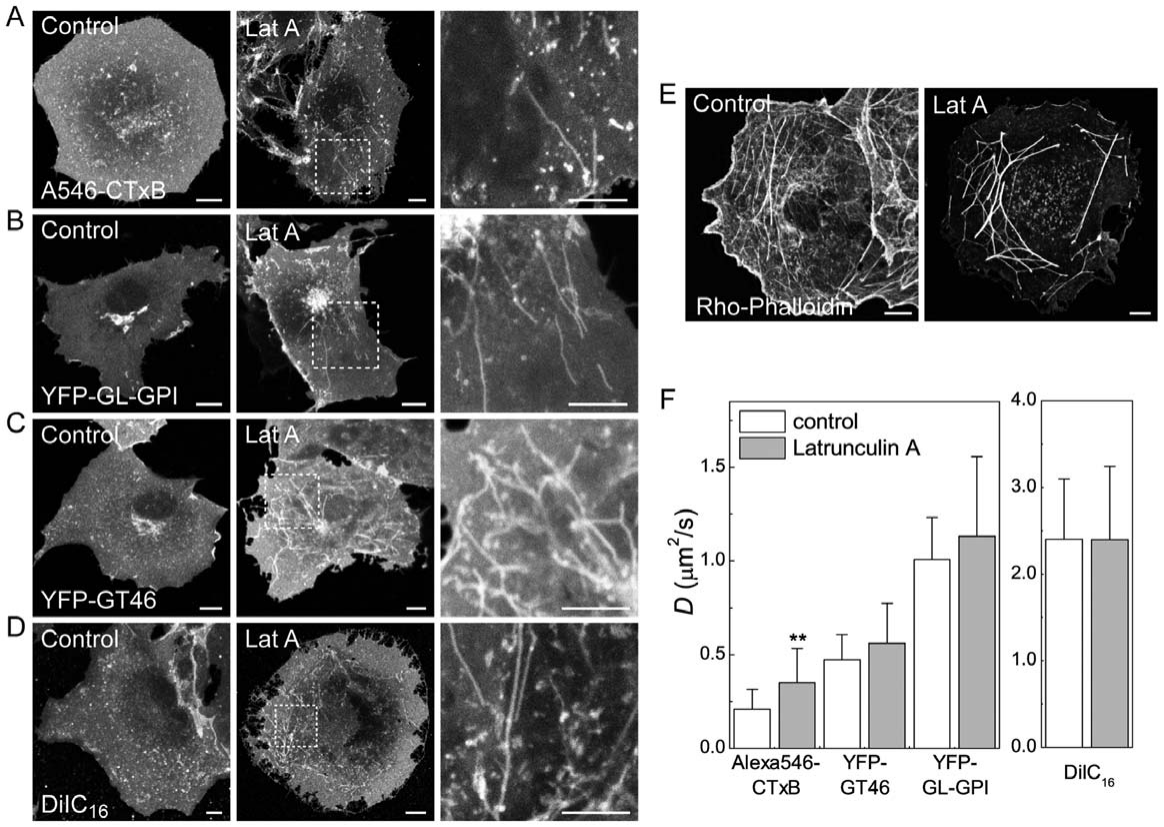
CTxB diffusion is confined by the actin cytoskeleton. (**A–D**) Subcellular distribution of (A) Alexa546-CTxB, (B) YFP-GL-GPI, (C) YFP-GT46 and (D) DiIC_16_ in live COS-7 cells under control conditions or following LatA treatment as described in the Material and Methods. A zoom of the boxed area in LatA treated cells is shown on the right for each marker. (**E**) Rhodamine phallodin staining in fixed COS-7 cells under control conditions or following LatA treatment. Bar, 10 µm. (**F**) COS-7 cells were treated with 1 µM LatA or mock-treated with 0.1% DMSO (“control”) for 5 minutes prior to imaging, and FRAP analysis was performed in the continued presence of LatA. Diffusion coefficients were measured for Alexa546-CTxB, YFP-GL-GPI, YFP-GT46 and DiIC_16_ in control and LatA treated COS-7 cells at 37°C (mean ± SD for 13–23 cells). ** *p*<0.01, Student t-test.

In control experiments, we confirmed that LatA treatment led to a loss of F-actin within cells as assessed by phalloidin staining of fixed cells, as expected (Figure 2E). Disruption of actin has also been shown previously to lead to the formation of tubular invaginations of the plasma membrane [48]. We verified that similar tubules were apparent in living cells labeled with CTxB or DiIC_16_, as well as in cells expressing YFP-GL-GPI or YFP-GT46 (Figure 2A–D).

Next, we used confocal FRAP to measure the diffusional mobility of CTxB in LatA-treated and mock-treated cells incubated in media containing DMSO (Figure 2F). For comparison, we monitored the effects of these treatments on the diffusional mobility of YFP-GL-GPI, YFP-GT46, and DiIC_16_ under identical conditions, avoiding regions where plasma membrane tubules were present in these measurements. The results of these experiments showed *D* for CTxB was significantly increased by LatA treatment from 0.21±0.10 µm^2^/s to 0.35±0.18 µm^2^/s. In contrast, the diffusion of YFP-GL-GPI, YFP-GT46, and DiIC_16_ was unaffected in the presence of LatA (Figure 2F). These results suggest the diffusional mobility of CTxB is selectively slowed either directly or indirectly as the result of its interactions with the actin cytoskeleton. However, even in cells in which actin was disrupted, the diffusion of CTxB was considerably slower than that of other cell surface molecules, suggesting additional factors are involved in slowing its lateral diffusion. We therefore asked if the interaction of CTxB with other types of domains might impede its mobility.

### Diffusion of CTxB and a transmembrane protein, but not a GPI-anchored protein is enhanced in ATP-depleted cells

Previous studies have reported that certain proteins associate with nanoclusters that maintain a fixed size and a fixed ratio of monomeric to clustered molecules over a wide range of concentrations, implying these domains are actively maintained and require cellular energy for their generation [42], [49]. GPI-anchored proteins are immobilized within these nanoclusters, indicating these domains have the capacity to impact the dynamics of cell surface molecules [29]. It is currently unknown if CTxB associates with actively maintained nanoclusters. We reasoned that ATP depletion might disrupt the interactions of molecules with such structures, thereby leading to an increase in their overall diffusional mobility. To test this, COS-7 cells were depleted of ATP by a 15 minute incubation with 0.02% sodium azide and 50 mM 2-deoxy-D-glucose, or mock-depleted prior to labeling with CTxB. For comparison, we performed similar experiments in ATP-depleted cells expressing YFP-GL-GPI (which is predicted to associate with nanoclusters) or YFP-GT46, or labeled with DiIC_16_.

**Figure 3:**
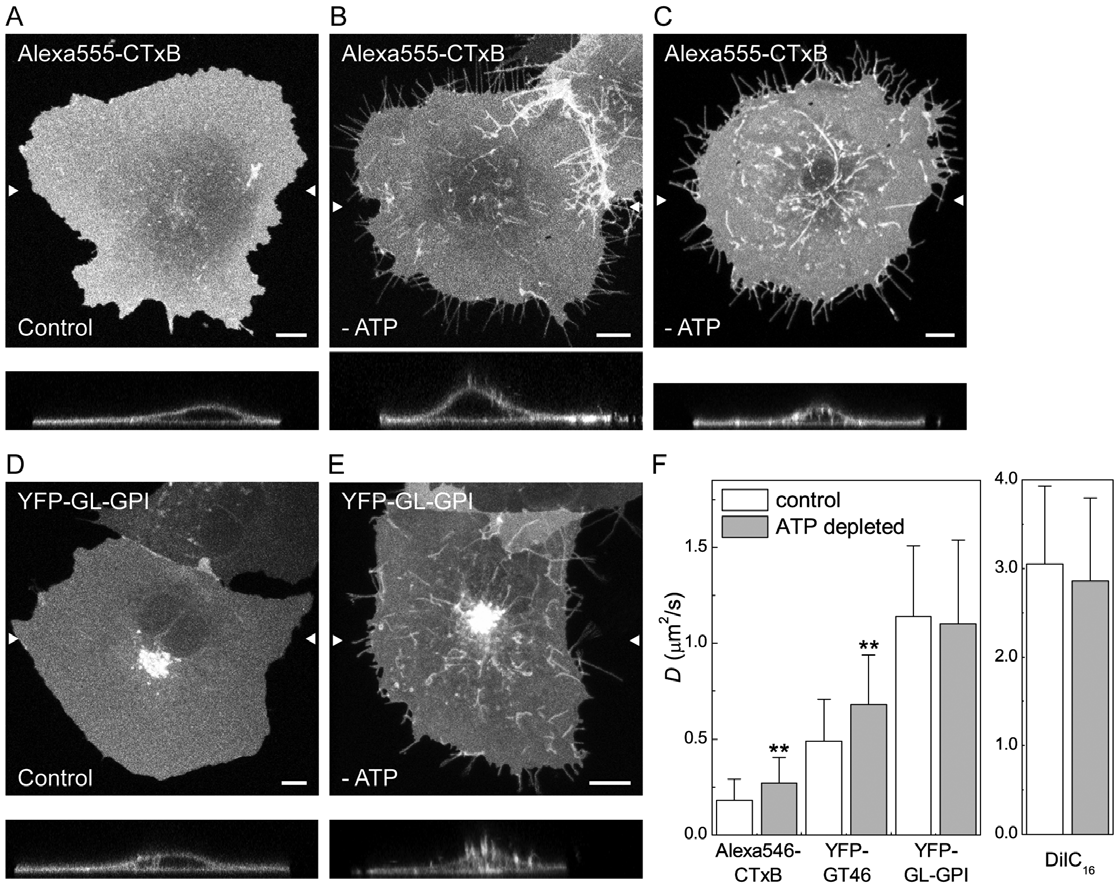
CTxB diffusion is confined by ATP-dependent barriers. (**A–E**) Subcellular distribution of Alexa555-CTxB and YFP-GL-GPI in control and ATP-depleted COS-7 cells. Images show the projection of a series of confocal slices through live cells. Arrows mark the position of an xz-section (shown below.) Scale bar = 10 µm. (A, D) Typical morphology of cells labeled with Alexa555-CTxB or expressing YFP-GL-GPI under control conditions. (B, E) In ATP depleted cells, in addition to labeling the bulk of the plasma membrane, Alexa555-CTxB and YFP-GL-GPI label protrusions of the plasma membrane found close to the coverslip, as well as protrusions projecting above the surface of the cells into the media. (C) Example of an ATP depleted cell in which CTxB accumulates in tubular plasma membrane invaginations in addition to protrusions. (**F**) COS-7 cells were ATP depleted or mock-depleted (“control”) for 15 minutes prior to labeling and FRAP was performed in the continued presence of ATP depletion or control medium. Diffusion coefficients were measured for Alexa546-CTxB, YFP-GT46, YFP-GL-GPI or DiIC_16_ (mean ± SD from 24–32 cells). Cells were labeled with 1 µM Alexa546-CTxB. FRAP was performed at 37°C. ** *p*<0.01, Student t-test.

**Figure 4:**
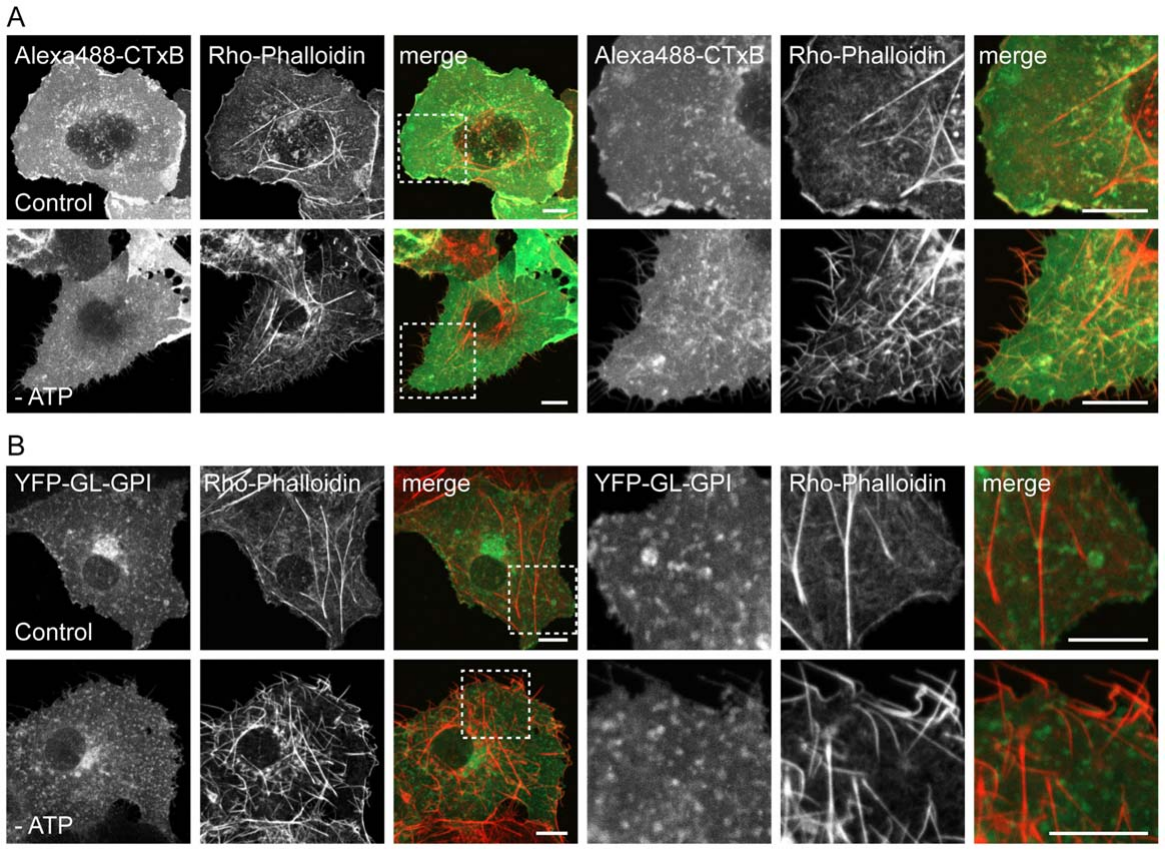
ATP depletion induces actin polymerization. Rhodamine-phalloidin labeling in mock-depleted and ATP depleted COS-7 cells (**A**) labeled with Alexa488-CTxB or (**B**) expressing YFP-GL-GPI. A single confocal section is shown for each. A zoom of the boxed area is shown on the right. The merged images show phalloidin staining in red and CTxB or YFL-GL-GPI staining in green. The spotty appearance of CTxB and YFP-GL-GPI is the result of fixation and permeabilization conditions. Scale bar = 10 µm.

ATP depletion led to several marked changes in the morphology of the plasma membrane and the underlying cytoskeleton. First, ATP depletion led to the formation of protrusions of the plasma membrane that were never observed in control cells (Figure 3A–E). Some of these protrusions were localized to the edges of cells and may represent retraction fibers. Needle-like protrusions were also seen projecting above cells into the media, as visualized in x-z sections. These protrusions were labeled with CTxB and for the other cell surface markers such as YFP-GL-GPI (Figure 3B, C, E). Second, in some ATP depleted cells tubular invaginations of the plasma membrane enriched in CTxB were observed (Figure 3C). Similar invaginations have been proposed to correspond to sites of clathrin-independent endocytosis induced by Shiga toxin B-subunit binding that tubulate in an ATP independent manner but whose scission is ATP dependent [7], [8], and have also been reported to form in cells labeled with CTxB [50]. Tubular invaginations were only observed in cells labeled with CTxB and not other cell surface markers, although the extent of invagination formation varied between cells. Because ATP depletion has been previously reported to increase levels of F-actin in cells [51]–[53], we also examined actin organization under these conditions by phalloidin staining. We found that F-actin staining was markedly enhanced in ATP-depleted cells (Figure 4). In addition, the plasma membrane protrusions also appeared to be enriched in actin, suggesting that the changes in actin organization that occur in response to ATP depletion may be responsible for their formation.

We next performed confocal FRAP analysis in ATP depleted cells. For these studies, we chose bleach ROIs on regions of the plasma membrane that did not include visible membrane protrusions or invaginations. The results of these experiments showed no change in *D* for YFP-GL-GPI or DiIC_16_ in ATP depleted versus mock-depleted cells (Figure 3F). We did note however that the rate of diffusion of DiIC_16_ in both mock ATP depleted and ATP depleted cells was significantly higher than that of DiIC_16_ under any of the other conditions examined. This effect was reproducible across days, and therefore likely arises from differences in the media used for various treatments.

In contrast to the lack of effect of ATP depletion on DiIC_16_ or YFP-GL-GPI, CTxB and YFP-GT46 both showed a significant increase in *D* in ATP depleted cells relative to controls (Figure 3F). We also observed a small but significant increase in M_f_ for DiIC_16_ and CTxB, and decrease in M_f_ for YFP-GT46 in ATP-depleted cells (Table S1). Thus, diffusion of CTxB is normally confined by an ATP-dependent mechanism. Because the diffusion of YFP-GL-GPI, which is predicted to associate with nanoclusters, was unaffected under these conditions, it seems unlikely that the increased mobility of CTxB is due to disruption of its association with actively maintained nanoclusters. Instead, the enhanced diffusion of CTxB under these conditions may reflect the substantial changes in the organization of actin that occur in response to ATP depletion (Figure 4), allowing for it to decouple from CTxB.

### Diffusion of CTxB and other cell surface molecules is identical in the presence and absence of caveolae

Caveolae are another structural feature of cell membranes with the potential to restrict the diffusion of CTxB. CTxB is sometimes enriched within caveolae, suggesting it has a specific affinity for these domains [30]–[33]. Since caveolae are immobile within the plane of the membrane [34], [35], even transient interactions of CTxB with caveolae would be expected to constrain its lateral mobility. In agreement with this possibility, several studies have reported that interactions of CTxB with caveolin-1 (Cav-1) itself slow the diffusion of CTxB both at the plasma membrane and within early endosomes at neutral pH [19], [31], [54]. However, these experiments either used a knockdown approach or examined the dynamics of CTxB in enlarged endosomes containing caveolin-1-GFP. Therefore, to more directly assess the effect of caveolae on CTxB mobility, we measured CTxB diffusion in Cav-1^−/−^ and Cav-1^+/+^ mouse embryonic fibroblast (MEF) cells.

**Figure 5:**
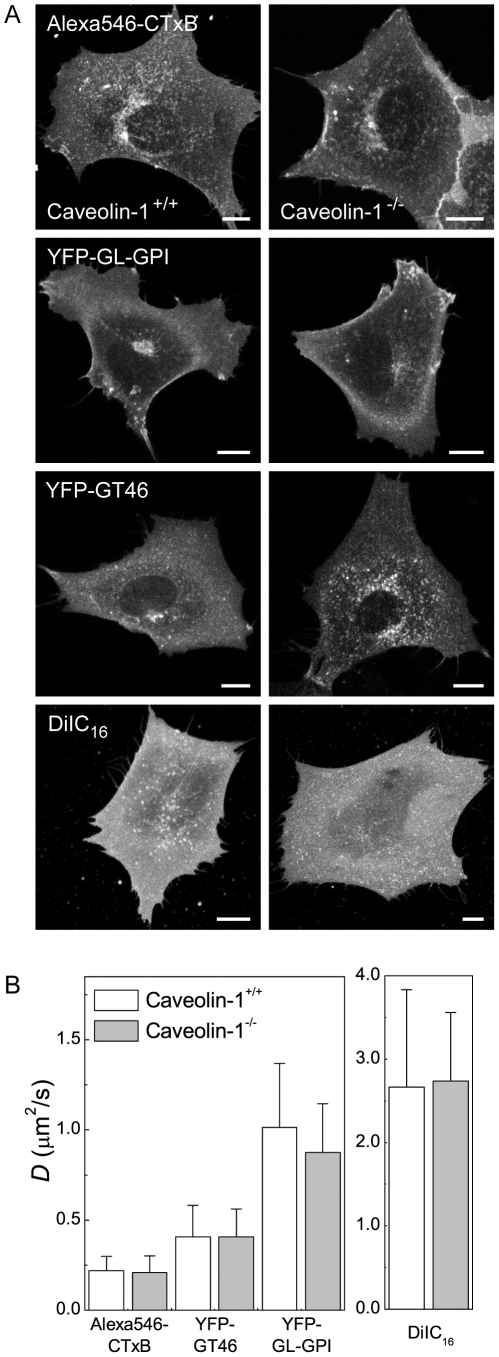
Caveolae have little effect on CTxB diffusion at the cell surface. (**A**) Subcellular distribution of Alexa546-CTxB, YFP-GL-GPI, YFP-GT46, and DiIC_16_ in live Cav-1^+/+^ and Cav-1^−/−^ MEF cells. Bar, 10 µm. (**B**) Diffusion coefficients of Alexa546-CTxB, YFP-GL-GPI, YFP-GT46 and DiIC_16_ in Cav-1^+/+^ and Cav-1^−/−^ MEF cells (mean ± SD from 22–47 cells). Cells were labeled with 1 µM Alexa546-CTxB. FRAP was performed at 37°C.

All of the markers studied localized correctly to the plasma membrane in the Cav-1^−/−^ cells (Figure 5A). Levels of CTxB binding to the cell surface were also equivalent in the Cav-1^+/+^ and Cav-1^−/−^ MEFs, similar to previous reports [55]. Confocal FRAP analysis revealed there was no significant difference in *D* or M_f_ between CTxB in Cav-1^+/+^ and Cav-1^−/−^ MEFs, suggesting that CTxB is not diffusionally trapped within caveolae (Figure 5B, Table S1). Furthermore, both the *D* and M_f_ values obtained for CTxB in MEFs were similar to those measured in COS-7 cells, which contain abundant caveolae, further suggesting that the slow diffusion of CTxB is not due to its interactions with caveolae. Analysis of the cell surface dynamics of YFP-GL-GPI, YFP-GT46, and DiIC_16_ also showed no differences in Cav-1^+/+^ and Cav-1^−/−^ cells (Figure 5B). These results suggest caveolae do not specifically confine the cell surface dynamics of CTxB, and also do not generally impact the diffusional mobility of other proteins or lipids.

### The diffusional mobility of other proteins and lipids at the cell surface is unaffected by the presence of bound CTxB

In the experiments described above, we focused on how structural components of the plasma membrane modulate the dynamics of CTxB. However, binding of CTxB to membranes could itself potentially alter the organization of the plasma membrane organization in a way that influences either its own diffusion, or that of other molecules. For example, binding of CTxB to cells could potentially create crowding effects that cause it to diffuse slowly [56]. Alternatively, the addition of CTxB to cells could lead to the formation of sub-resolution domains that influence the distribution and dynamics of other proteins and lipids, by analogy to its ability to form macroscopic domains in model systems [9], [10]. Cellular proteins with affinity for these domains might become trapped within or transiently interact with these structures, consequently slowing their diffusion as well [37]. Conversely, if such domains were sufficiently abundant and connected, other proteins could potentially become “trapped” within islands surrounded by a cluster of domains formed by CTxB binding [36]. Each of these models predicts that in the presence of CTxB, diffusion of other proteins and lipids should be slowed compared to the absence of CTxB.

**Figure 6:**
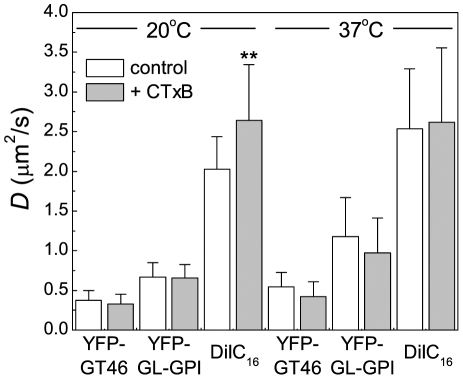
CTxB binding has little effect on the diffusion of other cell surface molecules. COS-7 cells expressing YFP-GL-GPI or YFP-GT46, or stained with DiIC_16_ were labeled with 1 µM Cy5-CTxB (YFP-GL-GPI and YFP-GT46) or Alexa488-CTxB (DiIC_16_) for 5 minutes at room temperature and washed prior to FRAP studies. Diffusion coefficients were measured for YFP-GL-GPI, YFP-GT46, and DiIC_16_ in the presence and absence of 1 µM Cy5 CTxB (mean ± SD for n = 16–32 cells). FRAP data were collected at both 20°C and 37°C. ** *p*<0.01, Student t-test.

To test this, we measured the diffusional mobility of DiIC_16_, YFP-GL-GPI, and YFP-GT46 in the presence or absence of saturating levels of CTxB (Figure 6, Table S1). Experiments were carried out at both 22°C and 37°C in order to test for the presence of temperature-dependent membrane percolation threshold [36]. Interestingly, the diffusion of both proteins (YFP-GL-GPI and YFP-GT46) was unaltered by the addition of CTxB at both temperatures. DiIC_16_ diffusion was also unchanged in the presence of CTxB at 37°C, but was significantly increased at 22°C. This implies that if microdomains are formed upon binding of CTxB to the cell surface, they are not sufficiently abundant or large enough to influence the diffusion of our test proteins at physiological temperature. However, they do appear to influence the mobility of DiIC_16_ at lower temperatures, raising the possibility that CTxB binding may impact the viscosity or order of the membrane at lower temperatures, perhaps by perturbing underlying lipid organization [16].

### The diffusional mobility of AB_5_ toxins is not correlated with their capacity to cluster glycolipids

Our findings raise the question of whether the confined diffusion we observed for CTxB is a general feature of proteins with the intrinsic ability to cluster glycolipids. The B subunit of Shiga toxin (STxB) is another example of a bacterially derived toxin with a homopentameric structure that binds a glycolipid receptor (in this case, Gb3) [4]. While there is no apparent similarity in the amino acid sequences of these two proteins, their structures are highly homologous [4]. Importantly, STxB can bind up to 15 Gb_3_ molecules per homopentamer [57]. We therefore predicted that STxB would diffuse even more slowly than CTxB if the extent of glycolipid clustering is a major determinant of their cell surface dynamics.

**Figure 7:**
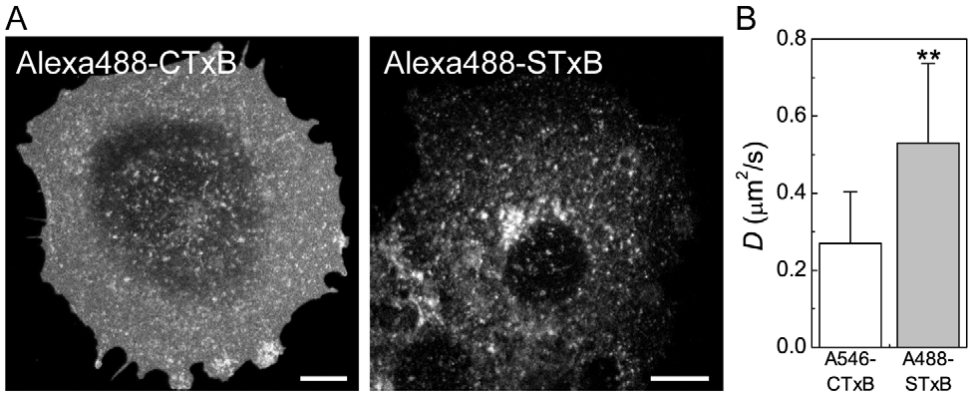
STxB, another homopentameric glycolipid-binding toxin, diffuses more rapidly than CTxB. (**A**) Subcellular distribution of CTxB and STxB in control cells approximately 5 minutes after labeling and shifting to 37°C. (**B**) Diffusion coefficients of STxB vs. CTxB in ATP depleted COS-7 cells (mean ± SD from 30 and 28 cells, respectively). Cells were labeled with 1 µM Alexa546-CTxB or 75 nM A488-STxB. FRAP was performed at 37°C. ** *p*<0.01, Student t-test.

To test this, we initially sought to measure the diffusional mobility of STxB in the plasma membrane of unperturbed COS-7 cells. We found that COS-7 cells normally label poorly with STxB. Therefore, to enable STxB binding, COS-7 cells were transfected with Gb3 synthase [58]. We next attempted to perform FRAP analysis of STxB at the cell surface. However, within minutes after labeling, STxB was rapidly internalized from the cell surface into numerous small, rapidly moving vesicles and tubular structures (Figure 7A), precluding FRAP analysis. We therefore took advantage of the fact that ATP depletion, a condition we used to study the regulation of the diffusion of CTxB (Figure 3), inhibits the internalization of STxB [7] and CTxB (this study) as a way to compare the dynamics of CTxB and STxB on the plasma membrane.

For these experiments, COS-7 cells expressing Gb_3_ synthase were preincubated in ATP depletion medium for 15 min prior to labeling with Alexa 488-STxB and subsequently imaged in the continued presence of ATP depletion medium. In ATP depleted cells, STxB often accumulated in tubular plasma membrane invaginations (data not shown), similar to those reported previously [7]. In some cells, STxB could also be found in protrusions induced by ATP depletion (data not shown), similar to those observed for other cell surface markers. Importantly, under these conditions, a substantial fraction of STxB remained trapped at the cell surface, enabling us to use confocal FRAP to assess the dynamics of the plasma membrane pool of the toxin. Remarkably, the cell surface pool of STxB diffused significantly faster than CTxB, with a characteristic *D* of ∼0.5 µm^2^/s and M_f_ of 81±7%. In fact, this was significantly faster than the diffusion of CTxB under any of the conditions we examined. These data indicate that confined diffusion is not a general property of glycolipid binding toxins, and suggest that in cells, the diffusional properties of CTxB and STxB are not correlated in a simple way with their capacity for clustering multiple glycolipids.

## Discussion

In contrast to the highly dynamic properties of lipids and many lipid-anchored proteins, CTxB diffuses extremely slowly when bound to its accessible pool of glycolipid receptors in the plasma membrane of living cells. In the current study, we analyzed the regulation of the dynamics of CTxB, with the goal of identifying mechanisms that confine the lateral diffusion of the CTxB/receptor complex.

To dissect how the cell surface dynamics of CTxB are controlled, we used a confocal FRAP assay [38], [39]. This technique can be used to quantitatively measure the diffusional mobility of a wide range of molecules in cells, from slowly diffusing membrane proteins like CTxB to rapidly diffusing proteins like soluble EGFP [38], [39]. An ensemble technique, confocal FRAP reports on the average rate of diffusion of a population of particles across micrometer distances, over timescales of seconds to minutes. Other approaches such as single particle tracking and fluorescence correlation spectroscopy, which report on the short-range diffusion of a small number of particles at one time, have also been used to study the diffusional mobility of CTxB. Like FRAP, these techniques also report that the diffusion of CTxB is highly confined [13], [17]–[22]. This indicates that the barriers that restrict the long-range motion of CTxB are conserved over smaller spatial and temporal scales.

We sought to understand how the cell surface dynamics of CTxB are regulated compared to a lipid-anchored protein, transmembrane protein, and lipid probe. Our results highlight several important differences in the behavior of these various classes of molecules. For example, although previous studies have indicated that the dynamics of many lipid-anchored molecules are unaffected by disruption of the actin cytoskeleton [27], actin plays an important role in constraining the diffusion of CTxB. The dependence of CTxB diffusion on actin organization may seem surprising given that CTxB binds a glycolipid receptor and thus lacks the ability to directly couple to actin. However, the CTxB/GM_1_ complex could potentially interact indirectly with actin in several ways. The diffusion of CTxB could be impeded by the presence of transmembrane “post” proteins that are attached to actin-based corrals [43], [59]. Alternatively, clustering of GM_1_ by CTxB could initiate signaling events that in turn transiently connect the CTxB/GM_1_ complex to actin with the help of currently unknown transmembrane proteins, by analogy to how crosslinked GPI-anchored proteins are thought to interact with actin [60]–[63]. The formation of a “textured” lipid phase in response to CTxB binding may contribute to signaling across the bilayer leaflets [16]. There is also evidence from freeze-fracture immunolabeling electron microscopy that GM_1_, the high affinity glycolipid receptor for CTxB, associates with actin-dependent clusters in cells [64]. Thus, GM_1_ itself may be coupled to actin, providing an indirect link between CTxB and the cytoskeleton.

Our results also indicate the diffusion of CTxB is normally confined by ATP-dependent processes. Initially, we set out to test the effects of this treatment as a way to assess the possible interaction of CTxB with actively maintained nanoclusters, a class of domains previously shown to lead to the local enrichment and immobilization of GPI-anchored proteins [29]. Despite the known interaction of GPI-anchored proteins with such structures, ATP depletion had little influence on the overall mobility of a representative protein, YFP-GL-GPI. We speculate this may be the case because only a relatively small fraction of GPI-anchored proteins associates with these domains [29]. However, ATP depletion also had a profound effect on actin organization and membrane structure. In particular, we observed a dramatic increase in F-actin staining close to the plasma membrane in response to ATP depletion, accompanied by the formation of needle-like protrusions. Based on these observations, we propose remodeling of actin to form longer filaments may increase the dimensions of the actin-defined compartments that normally confine protein and lipid diffusion at the cell surface [65], therefore increasing the mobility of CTxB in response to ATP depletion. This model might explain why ATP depletion and LatA treatment have similar effects on CTxB dynamics, even though they have much different effects on overall actin organization. Other changes in membrane structure and composition known to occur in response to ATP depletion, including inhibition of phosphoinositide synthesis and the release of some small GTPases from the plasma membrane [66], could also contribute to the shift in CTxB diffusion. This multiplicity of effects of ATP depletion may also explain why diffusion of the transmembrane protein YFP-GT46 was enhanced following ATP depletion, but unaffected by actin disruption following LatA treatment.

Our results suggest actin organization/dynamics alone are clearly not the only source of the low diffusion rate observed for CTxB, since even in the presence of LatA the bound toxin diffuses much more slowly than any of the other molecules examined. One possibility is that CTxB may recognize additional binding partners [67]–[69]. If one of these binding partners were a transmembrane protein, this could explain the slow diffusion of the toxin and would also provide a clear model for why CTxB diffusion is sensitive to cortical actin. It is also possible that CTxB diffusion is regulated by flotillin, which has been shown to modulate the diffusional mobility of other sphingolipid-binding molecules [70]. Because flotillin itself has been shown to interact with actin [71], it could also potentially serve to couple CTxB/GM_1_ complexes to the cytoskeleton.

Caveolae have been shown to become enriched in and internalize CTxB, although they are not required for its endocytic uptake into cells [55]. We therefore tested for a potential role of caveolae in controlling the overall diffusion of CTxB at the cell surface. We found that the absence of caveolae had no effect on the diffusion of CTxB, or for that matter on any of the other cell surface markers examined. *D* was also very similar for all the molecules examined in COS-7 cells (which contain caveolae) and in caveolin-1^−/−^ MEFs, further indicating that caveolae per se do not strongly influence the mobility of the molecules examined here. Taken together, we conclude from these studies that caveolae are not a major barrier to the diffusion of CTxB, and also do not function as general regulators of protein or lipid diffusion. This does not rule out the possibility that specific proteins or lipids interact with caveolae, especially following crosslinking [62]. Caveolae could also potentially become saturated with CTxB, as CTxB has been reported to be selectively taken up by caveolae when present at very low labeling concentrations [31]. The effects of caveolae and caveolin-1 on the diffusion of proteins like CTxB may also not necessarily be identical, since caveolin-1 can exist at the cell surface as small oligomers under conditions where caveolae per se are not present [19]. This may explain the difference between our current results and those of a previous study examining the effects of caveolin-1 on CTxB diffusion utilizing a knock down approach, that may have left these residual caveolin-1 oligomers on the cell surface [19], [54]. However, it is also formally possible that the role of caveolae and caveolin-1 in modulating the diffusion of CTxB are different in adipocytes and mammary tumor cells [19], [54] than in MEFs.

Because CTxB binding itself can potentially alter the organization of the plasma membrane by clustering glycolipids, we investigated its effects on the diffusion of other proteins and lipids. The results of these experiments showed very little change in protein or lipid diffusion in the presence of bound CTxB at physiological temperature. This result immediately rules out the possibility that CTxB binding causes crowding effects that slow its own diffusion [56]. They further imply that if CTxB forms small domains in intact cells, these domains do not incorporate either YFP-GL-GPI or YFP-GT46 [37], and also are not sufficiently large to form barriers to the diffusion of other proteins or lipids [36]. Our observation that CTxB binding does not alter the diffusion of YFP-GL-GPI is consistent with a recent near field scanning microscopy study showing that GPI-anchored proteins are in close proximity to CTxB, but do not directly colocalize with CTxB-enriched domains [72]. They somewhat differ, however, from data reported by Pinaud and colleagues [73]. In that study, the effects of CTxB on the diffusional mobility of an artificial GPI-anchored protein consisting of avidin attached to the membrane via a GPI-anchor, Av-GPI, were investigated in some detail using single quantum dot tracking [73]. A modest decrease in mobility of a slowly diffusing population of Av-GPI was reported to occur in cells labeled with CTxB. However, under steady state conditions, even the “fast” values of *D* reported for Av-GPI are almost two orders of magnitude slower than our measured *D* for YFP-GL-GPI (0.038 µm^2^/s for Av-GPI versus ∼1 µm^2^/s for YFP-GL-GPI). It thus seems likely that Pinaud et al. detected interactions of CTxB with a subset of partially immobilized GPI-anchored proteins, rather than a freely diffusing population of GPI-anchored proteins.

To better understand how the diffusional mobility of CTxB depends on its ability to cluster glycolipids, we compared its diffusion to that of the B-subunit of Shiga toxin, STxB. While the structures of these two toxins are very similar and therefore should have similar hydrodynamic radii, the fact that STxB binds 3 fold more lipids that CTxB would predict that STxB will have a stronger potential for cross-linking glycolipids than CTxB. However, in ATP depleted cells, the diffusion of STxB was *faster* than that of CTxB, suggesting that neither the size of the CTxB/GM_1_ complex nor the extent of glycosphingolipid clustering are the cause of CTxB's slow diffusion. The lack of correlation between the number of bound lipids and the rate of diffusion between these toxins mirrors a previous study in which we examined the role of crosslinking by comparing the diffusion of wild type CTxB to that of a chimeric form of cholera toxin with a mutant B-subunit containing only 1 or 2 GM_1_ binding sites instead of its usual 5 [13]. In that study, we found only a small difference in the rate of diffusion between wild type CTxB and mutant cholera toxin on the plasma membrane of COS-7 cells. Thus, it appears that the number of glycolipids bound by AB_5_ toxins has little effect on their cell surface dynamics. In addition, confined diffusion does not appear to be a conserved feature of these toxins.

We found that several of the treatments we examined, such as ATP depletion, caused detectable changes in the topology of the plasma membrane. Because the presence of surface roughness can cause simple diffusion processes to be underestimated [74], these topological changes alone could in principle lead to significant changes in diffusion. If this were the case, we would have expected to observe similar effects of these treatments on all of the cell surface molecules studied. Our results indicate that instead, the effects of these treatments most strongly altered the diffusional mobility of CTxB. Therefore, it seems unlikely that potential subresolution changes in cell surface topology accounted for the changes we observed in CTxB diffusion; rather, we propose these differences in diffusion were the result of lateral heterogeneity not directly related to cell surface topology. Further work is certainly needed to examine the interplay between membrane topology and lateral heterogeneity, as well as membrane topology and diffusion.

Until recently, it was assumed that GM_1_ levels at the cell surface control the extent of CTxB binding. Recent studies now indicate that the local microenvironment of glycosphingolipids is an important determinant of their accessibility to toxin binding, and CTxB binding thus cannot be considered as a reporter of all of the GM_1_ present in the plasma membrane [14], [15]. Interestingly, cholesterol depletion was shown to increase the levels of CTxB binding to cells. This suggests the masked fraction of GM_1_, rather than the fraction of GM_1_ normally accessible to CTxB, may be intrinsically associated with cholesterol-dependent membrane domains. The published literature on CTxB and related glycolipid-binding toxins such as STxB will need to be re-interpreted in light of these findings. In the current study, we did not attempt to separate out the effects of CTxB bound to different classes of GM_1_, instead focusing on the properties of complex formed upon binding of CTxB to the accessible population of GM_1_. In principle, one way to compare the properties of the toxin-accessible and toxin-inaccessible pool of GM_1_ would be to examine the effects of cholesterol depletion after unmasking the inaccessible pool by cholesterol depletion. However, cholesterol depletion itself can have profound effects on membrane structure. For example, in previous work we showed that cholesterol depletion using methyl-ß-cyclodextrin leads to a systematic slowing of the diffusion of multiple cell surface markers, including CTxB [22]. Thus, further work will be required to decouple the effects of cholesterol on glycosphingolipid masking, toxin accessibility, and the dynamics of specific CTxB/GM_1_ complexes.

In conclusion, our results are consistent with a model in which in cells, the diffusional mobility of CTxB/GM_1_ complexes is restricted by F-actin dependent as well as ATP-dependent processes, which may also be linked to the maintenance of actin organization. Coupling of these complexes to actin could potentially occur either with the help of currently unidentified proteins, or by trapping of small CTxB-enriched domains within actin-defined corrals. Indirect interactions of CTxB with the cytoskeleton could in turn provide a mechanism that facilitates toxin uptake by clathrin-independent endocytic mechanisms [8], [47].

## Materials and Methods

### Cells and reagents

COS-7 cells, caveolin-1^+/+^ mouse embryonic fibroblasts (MEF), and caveolin-1^−/−^ MEFs were acquired from ATCC (Manassas, VA). Cell lines were maintained in Dulbecco's modified Eagle medium (DMEM) containing 10% fetal bovine serum at 37°C and 5% CO_2_. Cells were plated on coverslips two days prior to experiments.

Cholera toxin B subunit from *Vibrio cholerae* (Sigma-Aldrich, St. Louis, MO) was labeled with Alexa546 using an Alexa546 fluorophore protein labeling kit (Invitrogen, Carlsbad, CA). Cy5-CTxB was produced using Cy5 monoreactive dye packs (Amersham Bioscience, Piscataway, NJ). Alexa488-CTxB, Alexa555-CTxB and DiIC_16_ (1,1′-dihexadecyl-3,3,3′,3′-tetramethylindocarbocyanine perchlorate) were obtained from Invitrogen (Carlsbad, CA). Rhodamine phalloidin was from Invitrogen-Molecular Probes (Carlsbad, CA). Yellow fluorescent protein (YFP) tagged versions of a model GPI-anchored protein (YFP-GL-GPI) and transmembrane protein (YFP-GT46) have been previously described [22], [40], [41]. Alexa488-STxB and a plasmid encoding Gb_3_ synthase [58], [75] were gifts from Dr. Ludger Johannes (Institut Curie, Paris, France). Transfections were performed 24 hours prior to imaging using FuGENE 6 as per the manufacturers instructions (Roche Diagnostics, Indianapolis, IN).

### Cell labeling

Cells were rinsed twice with imaging buffer (composed of phenol red-free DMEM supplemented with 25 mM HEPES (Sigma-Aldrich), and 1 mg/ml albumin bovine serum (Sigma-Aldrich)), and then incubated for 5 minutes at room temperature with the indicated concentration of CTxB (1 nM–1 µM), 5 µg/ml DiIC_16_ or 75 nM STxB. Cells were then rinsed twice with imaging buffer and imaged. For phallodin staining, cells were fixed in 3.7% PFA for 15 min at RT. They were then washed, permeabilized with 0.1% saponin in PBS containing 1 mg/ml bovine serum albumin for 15 min at room temperature (RT), and labeled with rhodamine-phalloidin (1∶40) for 30 min at RT, washed, and mounted in Fluoromount G (Southern Biotech, Birmingham, AL) supplemented with 25 mg/ml DABCO (1,4 diazabicyclo[2.2.2]octane) (Sigma-Aldrich) and allowed to solidify overnight prior to imaging.

### Actin depolymerization

Cells were washed with imaging buffer, incubated for 5 minutes in either 1 µM Alexa546-CTxB or 5 µg/ml DiIC_16_ in imaging buffer, and washed again. Actin depolymerization was then performed by incubating the cells at 37°C for 5 min in imaging buffer containing 1 µM Latrunculin A (LatA) (Sigma-Aldrich). Control cells were incubated in imaging buffer containing 0.1% DMSO. Cells were maintained in their respective buffer during imaging and all imaging was performed within 30 minutes of treatment. Alternatively, they were fixed and labeled with rhodamine phalloidin as indicated above.

### ATP depletion

ATP depletion was performed by pre-incubating cells at 37°C and 5% CO_2_ for 15 min in ATP depletion medium, composed of phenol-red free DMEM containing 50 mM 2-deoxy-D-glucose (Sigma-Aldrich), 0.02% sodium azide (Amersham Pharmacia Biotech), 25 mM HEPES (Sigma-Aldrich), and 1 mg/ml bovine serum albumin (Sigma-Aldrich)), as described in [76]. Control cells were incubated in ATP control medium (composed of phenol-red free DMEM with 50 mM D-(+)-glucose (Sigma-Aldrich), 25 mM HEPES, and 1 mg/ml BSA). Cells were then washed with their respective medium, incubated for 5 minutes in 1 µM Alexa546-CTxB, 5 µg/ml DiIC_16_ or 75 nM Alexa488-STxB (in either ATP depletion or control medium), washed again, and then imaged in the continued presence of ATP depletion or control medium. Imaging was completed within 45 minutes of labeling.

### Microscopy-based CTxB binding assay

1 µM stock of Alexa546-CTxB was prepared in imaging buffer and lower concentration stocks prepared by serial dilution. Cells were labeled with CTxB for 5 min at room temperature, washed, mounted live in imaging buffer, and visualized at 37°C. Images were taken on a Zeiss LSM 510 confocal microscope (Carl Zeiss Microimaging, Inc, Thornwood, N.Y.) with a 40X 1.4 NA Zeiss Plan-Neofluar objective at 0.7× zoom to acquire multiple cells per field. Fluorescence was excited at 543 nm with HeNe laser and detected with a preset Cy3 channel filter set provided by the manufacturer. The confocal pinhole was set at 2.17 Airy units. 512×512 pixel images were collected in 8 bit with line averaging of 8. Laser intensity and detector gain were set near pixel saturation for the 1 µM CTxB labeled cells and left unchanged across all concentrations. For lower concentrations duplicate images of the same field were taken at higher detector gain to validate ROI selection. Individual ROI's were drawn for each cell and for the background region, for images at matched laser intensity and detector gain, and mean pixel intensities in the ROI's collected using ImageJ (NIH, Bethesda, Maryland). Cell fluorescence was background corrected and then the mean and standard deviations computed for all cells at a given concentration.

### Confocal microscopy and confocal FRAP

Confocal microscopy and confocal FRAP experiments were carried out on a Zeiss LSM 510 confocal microscope (Carl Zeiss MicroImaging, Inc., Thornwood, NY) using a 40X 1.4 NA Zeiss Plan-Neofluar objective. Cells were maintained in phenol-red free DMEM containing 1 mg/ml albumin bovine serum and 50 mM HEPES supplemented with the indicated drugs for live-cell imaging experiments. Cells were maintained at 37°C using a stage heater and objective heater. EYFP and Alexa488 were excited using the 488 nm line of a 40 mW Argon laser, and Alexa546, Alexa555, rhodamine, or DiIC_16_ were excited at 543 nm of a HeNe laser and detected using filter sets provided by the manufacturer. For presentation purposes images were exported in tiff format and brightness and contrast was adjusted using Adobe Photoshop.

Z-sections were compiled using serial confocal images taken at 1 Airy unit. The confocal slices were taken with an optimal overlay of 0.46–0.48 µM. The images were collected using a 40X 1.4 NA Zeiss Plan-Neofluar objective with digital zoom of 2.2 to 3.0×. Images were collected either in 12 bit mode with line averaging of 4 or in 8 bit mode with line averaging of 8. To adjust for differences in cell size, the number of slices was varied while the degree of overlap between slices was held constant. Slices were then compiled into 3D projections and z-sections in LSM Image Browser (Carl Zeiss MicroImaging, Inc., Thornwood, NY).

For FRAP measurements, cells were imaged at 4X digital zoom with the confocal pinhole set between 1.01 and 1.99 Airy units. Full frame (512×512 pixel) images were collected for FRAP analysis of CTxB, YFP-GL-GPI, YFP-GT46, and STxB. For DiIC_16_ FRAP experiments, the imaging window was reduced to a 4.1×8.1 µm rectangle to speed image acquisition. Photobleaching of a circular bleach region 4.1 µm in diameter was performed by repetitively scanning the bleach region 10 times using the 488 nm laser line or both the 488 nm and 514 nm laser lines at full power. Prebleach and postbleach images were collected at lower laser power (typically 3% transmission or less) with either no line averaging or with line averaging of 2. FRAP measurements were carried out at 22°C, or at 37°C using an objective heater and heated stage insert.

### FRAP data analysis

Confocal FRAP data analysis was performed using a recently described method that corrects for diffusion that occurs during the photobleaching event [38] assuming a free diffusion model (as opposed to anomalous diffusion or reaction-diffusion type behavior). FRAP analysis was carried out essentially as described in [39], except that FRAP curves and post bleach intensity profiles were analyzed individually and that prior to fitting, FRAP curves were corrected for photobleaching during imaging by normalizing to the whole cell fluorescence over time as described previously [77]. Datasets in which endocytic vesicles were observed to pass through the bleach region were discarded. Mobile fractions were calculated as described previously [22] using the average of the last three time points as F_∞_ and the average of the three prebleach images as F_o_. Statistics were calculated with a Student t-test using OriginPro 8.5 (OriginLab Corp; Northampton, MA).

## Supporting Information

Table S1 Mobile fractions (%) of Alexa546-CTxB, YFP-GT46, YFP-GL-GPI, and DiIC16 following various treatments.
(DOC)

## References

[1] Simons K, Ikonen E (1997). Functional rafts in cell membranes.. Nature.

[2] Simons K, Gerl MJ (2010). Revitalizing membrane rafts: new tools and insights.. Nat Rev Mol Cell Biol.

[3] Johannes L, Mayor S (2010). Induced domain formation in endocytic invagination, lipid sorting, and scission.. Cell.

[4] Pina DG, Johannes L (2005). Cholera and Shiga toxin B-subunits: thermodynamic and structural considerations for function and biomedical applications.. Toxicon.

[5] Kaiser HJ, Lingwood D, Levental I, Sampaio JL, Kalvodova L (2009). Order of lipid phases in model and plasma membranes.. Proc Natl Acad Sci U S A.

[6] Levental I, Lingwood D, Grzybek M, Coskun U, Simons K (2010). Palmitoylation regulates raft affinity for the majority of integral raft proteins.. Proc Natl Acad Sci U S A.

[7] Römer W, Berland L, Chambon V, Gaus K, Windschiegl B (2007). Shiga toxin induces tubular membrane invaginations for its uptake into cells.. Nature.

[8] Römer W, Pontani LL, Sorre B, Rentero C, Berland L (2010). Actin dynamics drive membrane reorganization and scission in clathrin-independent endocytosis.. Cell.

[9] Lingwood D, Ries J, Schwille P, Simons K (2008). Plasma membranes are poised for activation of raft phase coalescence at physiological temperature.. Proc Natl Acad Sci U S A.

[10] Hammond AT, Heberle FA, Baumgart T, Holowka D, Baird B (2005). Crosslinking a lipid raft component triggers liquid ordered-liquid disordered phase separation in model plasma membranes.. Proc Natl Acad Sci U S A.

[11] Bacia K, Schwille P, Kurzchalia T (2005). Sterol structure determines the separation of phases and the curvature of the liquid-ordered phase in model membranes.. Proc Natl Acad Sci U S A.

[12] Lencer WI, Saslowsky D (2005). Raft trafficking of AB5 subunit bacterial toxins.. Biochim Biophys Acta.

[13] Wolf AA, Jobling MG, Saslowsky DE, Kern E, Drake KR (2008). Attenuated endocytosis and toxicity of a mutant cholera toxin with decreased ability to cluster GM1.. Infect Immun.

[14] Lingwood D, Binnington B, Rog T, Vattulainen I, Grzybek M (2011). Cholesterol modulates glycolipid conformation and receptor activity.. Nat Chem Biol.

[15] Mahfoud R, Manis A, Binnington B, Ackerley C, Lingwood CA (2010). A major fraction of glycosphingolipids in model and cellular cholesterol containing membranes are undetectable by their binding proteins.. J Biol Chem.

[16] Watkins EB, Miller CE, Majewski J, Kuhl TL (2011). Membrane texture induced by specific protein binding and receptor clustering: active roles for lipids in cellular function.. Proc Natl Acad Sci U S A.

[17] Bacia K, Scherfeld D, Kahya N, Schwille P (2004). Fluorescence correlation spectroscopy relates rafts in model and native membranes.. Biophys J.

[18] Hebbar S, Lee E, Manna M, Steinert S, Kumar GS (2008). A fluorescent sphingolipid binding domain peptide probe interacts with sphingolipids and cholesterol-dependent raft domains.. J Lipid Res.

[19] Lajoie P, Partridge EA, Guay G, Goetz JG, Pawling J (2007). Plasma membrane domain organization regulates EGFR signaling in tumor cells.. J Cell Biol.

[20] Guo L, Zhou D, Pryse KM, Okunade AL, Su X (2010). Fatty acid 2-hydroxylase mediates diffusional mobility of raft-associated lipids, GLUT4 level and lipogenesis in 3T3-L1 adipocytes.. J Biol Chem.

[21] Jaqaman K, Kuwata H, Touret N, Collins R, Trimble WS (2011). Cytoskeletal control of CD36 diffusion promotes its receptor and signaling function.. Cell.

[22] Kenworthy AK, Nichols BJ, Remmert CL, Hendrix GM, Kumar M (2004). Dynamics of putative raft-associated proteins at the cell surface.. J Cell Biol.

[23] Day CA, Kenworthy AK (2009). Tracking microdomain dynamics in cell membranes.. Biochim Biophys Acta.

[24] Sahl SJ, Leutenegger M, Hilbert M, Hell SW, Eggeling C (2010). Fast molecular tracking maps nanoscale dynamics of plasma membrane lipids.. Proc Natl Acad Sci U S A.

[25] Mueller V, Ringemann C, Honigmann A, Schwarzmann G, Medda R (2011). STED nanoscopy reveals molecular details of cholesterol- and cytoskeleton-modulated lipid interactions in living cells.. Biophys J.

[26] Eggeling C, Ringemann C, Medda R, Schwarzmann G, Sandhoff K (2009). Direct observation of the nanoscale dynamics of membrane lipids in a living cell.. Nature.

[27] Lenne PF, Wawrezinieck L, Conchonaud F, Wurtz O, Boned A (2006). Dynamic molecular confinement in the plasma membrane by microdomains and the cytoskeleton meshwork.. EMBO J.

[28] Goodwin JS, Drake KR, Remmert CL, Kenworthy AK (2005). Ras diffusion is sensitive to plasma membrane viscosity.. Biophys J.

[29] Goswami D, Gowrishankar K, Bilgrami S, Ghosh S, Raghupathy R (2008). Nanoclusters of GPI-anchored proteins are formed by cortical actin-driven activity.. Cell.

[30] Pang H, Le PU, Nabi IR (2004). Ganglioside GM1 levels are a determinant of the extent of caveolae/raft-dependent endocytosis of cholera toxin to the Golgi apparatus.. J Cell Sci.

[31] Pelkmans L, Burli T, Zerial M, Helenius A (2004). Caveolin-stabilized membrane domains as multifunctional transport and sorting devices in endocytic membrane traffic.. Cell.

[32] Parton RG (1994). Ultrastructural localization of gangliosides; GM1 is concentrated in caveolae.. J Histochem Cytochem.

[33] Schnitzer JE, Oh P, McIntosh DP (1996). Role of GTP hydrolysis in fission of caveolae directly from plasma membranes [published erratum appears in Science 1996 Nov 15;274(5290):1069].. Science.

[34] Thomsen P, Roepstorff K, Stahlhut M, van Deurs B (2002). Caveolae are highly immobile plasma membrane microdomains, which are not involved in constitutive endocytic trafficking.. Mol Biol Cell.

[35] Pelkmans L, Puntener D, Helenius A (2002). Local actin polymerization and dynamin recruitment in SV40-induced internalization of caveolae.. Science.

[36] Meder D, Moreno MJ, Verkade P, Vaz WL, Simons K (2006). Phase coexistence and connectivity in the apical membrane of polarized epithelial cells.. Proc Natl Acad Sci U S A.

[37] Shvartsman DE, Kotler M, Tall RD, Roth MG, Henis YI (2003). Differently anchored influenza hemagglutinin mutants display distinct interaction dynamics with mutual rafts.. J Cell Biol.

[38] Kang M, Day CA, Drake K, Kenworthy AK, DiBenedetto E (2009). A generalization of theory for two-dimensional fluorescence recovery after photobleaching applicable to confocal laser scanning microscopes.. Biophys J.

[39] Drake KR, Kang M, Kenworthy AK (2010). Nucleocytoplasmic distribution and dynamics of the autophagosome marker EGFP-LC3.. PLoS ONE.

[40] Keller P, Toomre D, Diaz E, White J, Simons K (2001). Multicolour imaging of post-Golgi sorting and trafficking in live cells.. Nat Cell Biol.

[41] Pralle A, Keller P, Florin EL, Simons K, Horber JK (2000). Sphingolipid-cholesterol rafts diffuse as small entities in the plasma membrane of mammalian cells.. J Cell Biol.

[42] Sharma P, Varma R, Sarasij RC, Ira, Gousset K (2004). Nanoscale organization of multiple GPI-anchored proteins in living cell membranes.. Cell.

[43] Kusumi A, Nakada C, Ritchie K, Murase K, Suzuki K (2005). Paradigm shift of the plasma membrane concept from the two-dimensional continuum fluid to the partitioned fluid: high-speed single-molecule tracking of membrane molecules.. Annu Rev Biophys Biomol Struct.

[44] Kusumi A, Sako Y (1996). Cell surface organization by the membrane skeleton.. Curr Opin Cell Biol.

[45] Marguet D, Lenne PF, Rigneault H, He HT (2006). Dynamics in the plasma membrane: how to combine fluidity and order.. EMBO J.

[46] Andrews NL, Lidke KA, Pfeiffer JR, Burns AR, Wilson BS (2008). Actin restricts FcepsilonRI diffusion and facilitates antigen-induced receptor immobilization.. Nat Cell Biol.

[47] Badizadegan K, Wheeler HE, Fujinaga Y, Lencer WI (2004). Trafficking of cholera toxin-ganglioside GM1 complex into Golgi and induction of toxicity depend on actin cytoskeleton.. Am J Physiol Cell Physiol.

[48] van Deurs B, von Bulow F, Vilhardt F, Holm PK, Sandvig K (1996). Destabilization of plasma membrane structure by prevention of actin polymerization. Microtubule-dependent tubulation of the plasma membrane.. J Cell Sci.

[49] Plowman SJ, Muncke C, Parton RG, Hancock JF (2005). H-ras, K-ras, and inner plasma membrane raft proteins operate in nanoclusters with differential dependence on the actin cytoskeleton.. Proc Natl Acad Sci U S A.

[50] Ewers H, Römer W, Smith AE, Bacia K, Dmitrieff S (2010). GM1 structure determines SV40-induced membrane invagination and infection.. Nat Cell Biol.

[51] Hinshaw DB, Burger JM, Miller MT, Adams JA, Beals TF (1993). ATP depletion induces an increase in the assembly of a labile pool of polymerized actin in endothelial cells.. Am J Physiol.

[52] Herget-Rosenthal S, Hosford M, Kribben A, Atkinson SJ, Sandoval RM (2001). Characteristics of EYFP-actin and visualization of actin dynamics during ATP depletion and repletion.. Am J Physiol Cell Physiol.

[53] Atkinson SJ, Hosford MA, Molitoris BA (2004). Mechanism of actin polymerization in cellular ATP depletion.. J Biol Chem.

[54] Gonzalez-Munoz E, Lopez-Iglesias C, Calvo M, Palacin M, Zorzano A (2009). Caveolin-1 loss of function accelerates glucose transporter 4 and insulin receptor degradation in 3T3-L1 adipocytes.. Endocrinology.

[55] Kirkham M, Fujita A, Chadda R, Nixon SJ, Kurzchalia TV (2005). Ultrastructural identification of uncoated caveolin-independent early endocytic vehicles.. J Cell Biol.

[56] Frick M, Schmidt K, Nichols BJ (2007). Modulation of lateral diffusion in the plasma membrane by protein density.. Curr Biol.

[57] Ling H, Boodhoo A, Hazes B, Cummings MD, Armstrong GD (1998). Structure of the shiga-like toxin I B-pentamer complexed with an analogue of its receptor Gb3.. Biochemistry.

[58] Steffensen R, Carlier K, Wiels J, Levery SB, Stroud M (2000). Cloning and expression of the histo-blood group Pk UDP-galactose: Ga1beta-4G1cbeta1-cer alpha1, 4-galactosyltransferase. Molecular genetic basis of the p phenotype.. J Biol Chem.

[59] Umemura YM, Vrljic M, Nishimura SY, Fujiwara TK, Suzuki KG (2008). Both MHC class II and its GPI-anchored form undergo hop diffusion as observed by single-molecule tracking.. Biophys J.

[60] Suzuki KG, Fujiwara TK, Edidin M, Kusumi A (2007). Dynamic recruitment of phospholipase C gamma at transiently immobilized GPI-anchored receptor clusters induces IP3-Ca2+ signaling: single-molecule tracking study 2.. J Cell Biol.

[61] Suzuki KG, Fujiwara TK, Sanematsu F, Iino R, Edidin M (2007). GPI-anchored receptor clusters transiently recruit Lyn and G alpha for temporary cluster immobilization and Lyn activation: single-molecule tracking study 1.. J Cell Biol.

[62] Chen Y, Thelin WR, Yang B, Milgram SL, Jacobson K (2006). Transient anchorage of cross-linked glycosyl-phosphatidylinositol-anchored proteins depends on cholesterol, Src family kinases, caveolin, and phosphoinositides.. J Cell Biol.

[63] Chen Y, Veracini L, Benistant C, Jacobson K (2009). The transmembrane protein CBP plays a role in transiently anchoring small clusters of Thy-1, a GPI-anchored protein, to the cytoskeleton.. J Cell Sci.

[64] Fujita A, Cheng J, Fujimoto T (2009). Segregation of GM1 and GM3 clusters in the cell membrane depends on the intact actin cytoskeleton.. Biochim Biophys Acta.

[65] Morone N, Fujiwara T, Murase K, Kasai RS, Ike H (2006). Three-dimensional reconstruction of the membrane skeleton at the plasma membrane interface by electron tomography.. J Cell Biol.

[66] Gomez GA, Daniotti JL (2007). Electrical properties of plasma membrane modulate subcellular distribution of K-Ras.. Febs J.

[67] Blank N, Schiller M, Krienke S, Wabnitz G, Ho AD (2007). Cholera toxin binds to lipid rafts but has a limited specificity for ganglioside GM1.. Immunol Cell Biol.

[68] Yanagisawa M, Ariga T, Yu RK (2006). Cholera toxin B subunit binding does not correlate with GM1 expression: a study using mouse embryonic neural precursor cells.. Glycobiology.

[69] Hansen GH, Dalskov SM, Rasmussen CR, Immerdal L, Niels-Christiansen LL (2005). Cholera toxin entry into pig enterocytes occurs via a lipid raft- and clathrin-dependent mechanism.. Biochemistry.

[70] Zhang D, Manna M, Wohland T, Kraut R (2009). Alternate raft pathways cooperate to mediate slow diffusion and efficient uptake of a sphingolipid tracer to degradative and recycling compartments.. J Cell Sci.

[71] Langhorst MF, Solis GP, Hannbeck S, Plattner H, Stuermer CA (2007). Linking membrane microdomains to the cytoskeleton: regulation of the lateral mobility of reggie-1/flotillin-2 by interaction with actin.. FEBS Lett.

[72] van Zanten TS, Gomez J, Manzo C, Cambi A, Buceta J (2010). Direct mapping of nanoscale compositional connectivity on intact cell membranes.. Proc Natl Acad Sci U S A.

[73] Pinaud F, Michalet X, Iyer G, Margeat E, Moore HP (2009). Dynamic partitioning of a glycosyl-phosphatidylinositol-anchored protein in glycosphingolipid-rich microdomains imaged by single-quantum dot tracking.. Traffic.

[74] Adler J, Shevchuk AI, Novak P, Korchev YE, Parmryd I (2010). Plasma membrane topography and interpretation of single-particle tracks.. Nat Methods.

[75] Tetaud C, Falguieres T, Carlier K, Lecluse Y, Garibal J (2003). Two distinct Gb3/CD77 signaling pathways leading to apoptosis are triggered by anti-Gb3/CD77 mAb and verotoxin-1.. J Biol Chem.

[76] Nehls S, Snapp EL, Cole NB, Zaal KJ, Kenworthy AK (2000). Dynamics and retention of misfolded proteins in native ER membranes.. Nat Cell Biol.

[77] Kenworthy AK, McIntosh T (2007). Fluorescence recovery after photobleaching studies of lipid rafts.. Lipid Rafts.

