# Supplementary material for: Mechanisms Underlying the Confined Diffusion of Cholera Toxin B-Subunit in Intact Cell Membranes
Source: PLoS One. 2012 Apr 12;7(4):e34923. doi: 10.1371/journal.pone.0034923 (PMC3325267; doi:10.1371/journal.pone.0034923)
Supplement: Table S1 — Mobile fractions (%) of Alexa546-CTxB, YFP-GT46, YFP-GL-GPI, and DiIC16 following various treatments. (DOC) [file pone.0034923.s001.doc]

| **Table S1. Mobile fractions (%) of Alexa546-CTxB, YFP-GT46, YFP-GL-GPI, and DiIC_16_ following various treatments** | | | | | | | | | | |
| --- | --- | --- | --- | --- | --- | --- | --- | --- | --- | --- |
|  | **− CTxB** | | **+ CTxB** | | **ATP control** | **− ATP** | **Lat A control** | **+ Lat A** | **Cav-1^+/+^** | **Cav-1^-/-^** |
|  | **20°C** | **37°C** | **20°C** | **37°C** | **20°C** | **37°C** | **37°C** | **37°C** | **37°C** | **37°C** |
| **Alexa546-CTxB** | NA | NA | NA | NA | 79 ± 8 | 85 ± 8* | 81 ± 8 | 84 ± 7 | 82 ± 7 | 81 ± 7 |
|  |  |  |  |  | (30) | (30) | (23) | (19) | (24) | (26) |
| **YFP-GT46** | 90 ± 4 | 84 ± 6 | 92 ± 5 | 87 ± 6 | 83 ± 9 | 77 ± 7** | 81 ± 7 | 79 ± 7 | 85 ± 9 | 82 ± 10 |
|  | (23) | (22) | (16) | (17) | (24) | (29) | (22) | (19) | (23) | (22) |
| **YFP-GL-GPI** | 88 ± 8 | 89 ± 6 | 90 ± 6 | 91 ± 6 | 86 ± 6 | 85 ± 9 | 85 ± 7 | 81 ± 10 | 88 ± 7 | 85 ± 7 |
|  | (19) | (31) | (19) | (31) | (32) | (32) | (19) | (13) | (37) | (31) |
| **DiIC_16_** | 89 ± 5 | 89 ± 5 | 88 ± 5 | 90 ± 6 | 86 ± 5 | 90 ± 5** | 88 ± 3 | 89 ± 7 | 83 ± 7 | 83 ± 8 |
|  | (21) | (32) | (20) | (32) | (19) | (24) | (23) | (22) | (47) | (45) |
| Values represent mean ± SD. The numbers in parentheses represent sample size. | | | | | | | | | | |
| *****0.05 > *p* > 0.01; Student t-test against control at the same temperature | | | | | | | | | | |
| *******p* < 0.01; Student t-test against control at the same temperature | | | | | | | | | | |
